# Loss of the Rhodobacter capsulatus Serine Acetyl Transferase Gene, *cysE1*, Impairs Gene Transfer by Gene Transfer Agents and Biofilm Phenotypes

**DOI:** 10.1128/aem.00944-22

**Published:** 2022-09-13

**Authors:** David Sherlock, Paul C. M. Fogg

**Affiliations:** a Biology Department, University of York, York, United Kingdom; b York Biomedical Research Institute, University of York, York, United Kingdom; Washington University in St. Louis

**Keywords:** gene transfer agent, *Rhodobacter*, amino acid biosynthesis, bacteriophage evolution, biofilms, capsular polysaccharide, cysteine

## Abstract

Biofilms are widespread in the environment, where they allow bacterial species to survive adverse conditions. Cells in biofilms are densely packed, and this proximity is likely to increase the frequency of horizontal gene transfer. Gene transfer agents (GTAs) are domesticated viruses with the potential to spread any gene between bacteria. GTA production is normally restricted to a small subpopulation of bacteria, and regulation of GTA loci is highly coordinated, but the environmental conditions that favor GTA production are poorly understood. Here, we identified a serine acetyltransferase gene, *cysE1*, in Rhodobacter capsulatus that is required for optimal receipt of GTA DNA, accumulation of extracellular polysaccharide, and biofilm formation. The *cysE1* gene is directly downstream of the core *Rhodobacter*-like GTA (RcGTA) structural gene cluster and upregulated in an RcGTA overproducer strain, although it is expressed on a separate transcript. The data we present suggest that GTA production and biofilm are coregulated, which could have important implications for the study of rapid bacterial evolution and understanding the full impact of GTAs in the environment.

**IMPORTANCE** Direct exchange of genes between bacteria leads to rapid evolution and is the major factor underlying the spread of antibiotic resistance. Gene transfer agents (GTAs) are an unusual but understudied mechanism for genetic exchange that are capable of transferring any gene from one bacterium to another, and therefore, GTAs are likely to be important factors in genome plasticity in the environment. Despite the potential impact of GTAs, our knowledge of their regulation is incomplete. In this paper, we present evidence that elements of the cysteine biosynthesis pathway are involved in coregulation of various phenotypes required for optimal biofilm formation by Rhodobacter capsulatus and successful infection by the archetypal RcGTA. Establishing the regulatory mechanisms controlling GTA-mediated gene transfer is a key stepping stone to allow a full understanding of their role in the environment and wider impact.

## INTRODUCTION

Bacterial abundance in environmental water sources is routinely reported as millions to tens of millions of planktonic cells per mL (1–4). In addition, there are numerous abiotic and biotic surfaces available for bacterial attachment colonization. Biofilm formation is widespread in the environment and particularly in aquatic environments, where every solid surface is likely to be colonized by bacteria (5). Production of biofilm has numerous benefits, including protection from predation, resistance to starvation, and reduction of antibiotic susceptibility. Adherence to surfaces and establishment of biofilm architecture is known to be dependent on cell-cell communication and accumulation of cell-associated extracellular polysaccharides (EPS), DNA, and proteins. EPS itself is not usually essential for bacterial life; however, it is highly advantageous for survival and adaptation to challenging environments (6).

One of the major contributors to bacterial aquatic communities and biofilm is the alphaproteobacteria (7), of which *Rhodobacterales* species are key constituents (8, 9). Rhodobacter capsulatus is a metabolically versatile species capable of anaerobic photosynthetic and aerobic chemoheterotrophic growth (10). R. capsulatus biofilm production has not been extensively studied. However, one study has linked biofilm production to intracellular cyclic di-GMP (c-di-GMP) levels and the persulfide-responsive transcription factor SqrR (11). The EPS biosynthesis and export pathway has been identified, and this pathway is controlled by quorum sensing (12–14). EPS is also required for efficient gene transfer agent (GTA) binding to recipient cells in R. capsulatus (13, 15, 16).

GTAs are small virus-like particles that are produced by diverse species of bacteria and at least one archaeon (17), and they are thought to be descended from ancient bacteriophages that were domesticated by the host (18, 19). The genes encoding GTAs are embedded in the genomes of their host, often at multiple locations (20, 21), and coordinated expression is initiated from a small subset of the bacterial population (22–25). GTAs usually package and transfer “random” fragments of DNA from their host to compatible recipients (25–28), although Dinoroseobacter shibae GTAs do have biases to certain genomic regions (29). Unlike true viruses, GTAs do not preferentially transfer their own genes (25, 27). Timing and regulation of GTA production are tightly controlled by interlinked host regulatory circuits, including quorum sensing (14, 30), stringent response (31), SOS response (32), c-di-GMP (33, 34) and the pleiotropic transcription factor CtrA (35, 36). In *Rhodobacter*-like GTAs (RcGTAs), these complex pathways are integrated via a specific GTA transcriptional regulator, GafA (23), and an enigmatic secreted repressor encoded by *rcc00280* (37).

In the alphaproteobacteria, GTAs are predicted to be present in at least 50% of sequenced genomes where transmission appears to be via vertical inheritance (18, 38, 39). The ecological role of GTAs in the environment and the evolutionary benefit of GTA production are not currently known (38, 40–42); however, the only *in situ* study of GTA activity reported extraordinary gene transfer frequencies far in excess of other transfer mechanisms (43). Here, we explored the roles of the two genes located immediately downstream of the core R. capsulatus GTA gene cluster, *rcc01699* (referred to as *g16* here) and *rcc01700* (*cysE1*). Our findings indicate that *cysE1* plays an important role in the regulation of biofilm production and RcGTA receipt, and we propose that biofilms could provide an ideal environment for GTA activity.

## RESULTS

### Extension of the core RcGTA gene cluster.

**FIG 1:**
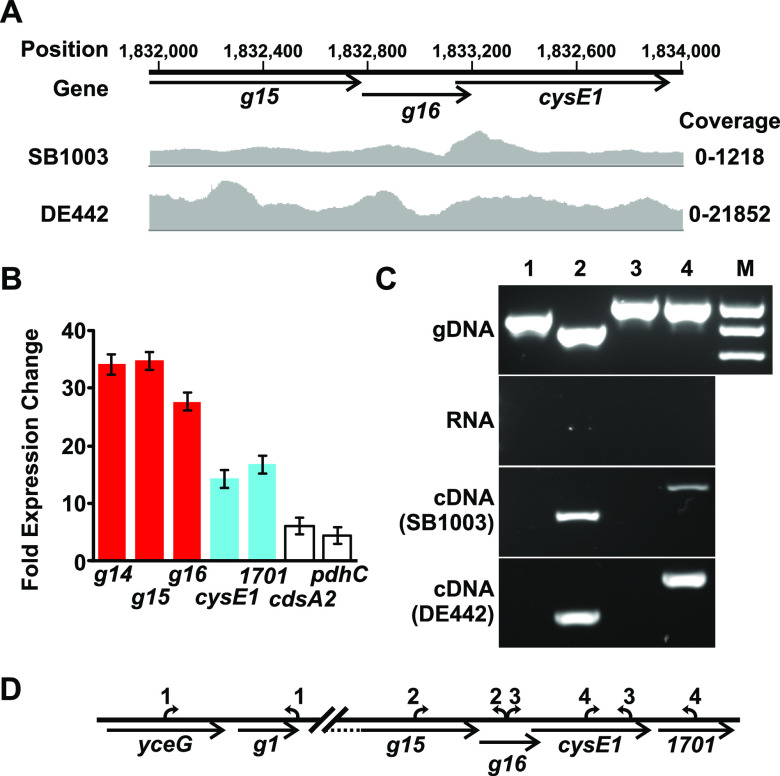
Defining the RcGTA transcriptional unit. (A) Representative histograms showing RNA-seq transcript coverage from the R. capsulatus wild-type strain SB1003 and the GTA overproducer DE442. Genome position (in base pairs) is indicated at the top, and the location of each gene is depicted with arrows beneath. The RNA-seq data set was described in reference 23, and the raw data can be found in the NCBI GEO Database, accession no. GSE118116. (B) Chart showing fold changes in gene expression between R. capsulatus DE442 (*n* = 8) and wild-type SB1003 (*n* = 4) in the GSE118116 data set. Red bars indicate constituents of the core RcGTA gene cluster, cyan shows the *cysE1* transcriptional unit, and white indicates the genes immediately downstream. Error bars represent standard deviations. (C) Agarose gel of a representative RT-PCR amplification. Templates were genomic DNA, DNA-free RNA, and cDNA isolated from SB1003 and DE442, as indicated. The 600- to 1,000-bp portion of the HyperLadder 1-kb DNA marker is included for reference (M) (Meridian Bioscience, Cincinnati, OH). PCR amplification occurred across the gene junctions indicated above each well and defined in panel D. (D) Schematic of the regions immediately before and after the RcGTA gene cluster. Primer pairs are numbered 1 to 4 and correspond to the wells shown in the preceding gel. The *yceG* gene immediately precedes the RcGTA small terminase gene, *g1*, which has previously been confirmed as the first gene of the operon and was used here as a negative control for genomic DNA carryover.

The core genes required for production of the R. capsulatus GTA particles (RcGTA), *rcc01682* to *rcc01698* (also known as *g1* to *g15*), cluster in an ~14.5-kb region that is flanked on either side by host metabolic genes (36). As the RcGTA genes are not excised during propagation, and there are no signature sequences (*att* sites) to indicate the precise boundaries of the RcGTA gene cluster, the true limits of the operon are unknown (17). Previous transcriptome data indicated that several genes downstream of the RcGTA gene cluster are upregulated in a RcGTA overproducer strain (R. capsulatus DE442) to levels comparable to those of the preceding RcGTA genes (Fig. 1) (23, 44).

The first downstream gene, *rcc01699* (or *g16*), begins 3 bases after the RcGTA *g15* stop codon. The *g16* gene encodes a hypothetical protein, and we were not able to assign a definitive predicted function through primary sequence or structural comparisons. The structure of g16 was predicted using the AlphaFold software (45), and the top-ranked model was submitted as a query to the Dali protein structure comparison server (see Table S1 in the supplemental material) (46). The best match was to the catalytic domain of the Bacillus cereus SleB protein (PDB code 4F55). SleB is a lytic transglycosylase that is involved in peptidoglycan remodeling during spore germination (47). It is conceivable that *g16* has a role in ingress or egress of RcGTAs during lysis or infection, but no experimental data have been produced so far to support this. The second downstream gene, *rcc01700* (*cysE1*), overlaps *g16* by 59 bp. CysE1 is a putative serine *O*-acetyltransferase, an enzyme that produces *O*-acetyl-l-serine (OAS) from acetyl coenzyme A (acetyl-CoA) and serine (48), an early step in the production of cysteine and methionine (49). Other than amino acid biosynthesis, OAS and its racemer *N*-acetyl-serine (NAS) have also been implicated as signaling molecules in various cellular processes, including biofilm production by other species (50–55).

Raw transcriptome sequencing (RNA-seq) reads obtained from the wild-type R. capsulatus SB1003 strain and the DE442 RcGTA overproducer data sets were mapped onto the *g15*-to-*cysE1* region of the genome, and no gaps in mRNA coverage were detected between the genes (Fig. 1A) (23). These data are consistent with a previous gene coexpression network analysis that indicated that *rcc01688* to *cdsA2* (*rcc01702*) form a single transcriptional unit (44). PCRs using cDNA template and primers located on either side of the RcGTA *g15*-*g16* and *cysE1*-*rcc01701* gene boundaries produced successful amplifications (Fig. 1C and D). However, no amplification was detected with primers spanning the *g16*-*cysE1* junction (Fig. 1C and D). Control amplification of a genomic DNA template using identical conditions successfully amplified all target sequences (Fig. 1C). These data indicate that *g16* is transcribed as part of the core RcGTA mRNA transcript and *cysE1* and *rcc01701* are located on a separate transcript, which is consistent with the relative expression levels observed in RNA-seq data (Fig. 1B) (23). Meanwhile, no amplification was detected across the boundary of the RcGTA small terminase gene (*rcc01682* [RcGTA *g1*]) and the gene immediately upstream (*yceG*), which has never been associated with RcGTA production or regulation (Fig. 1C). The data presented indicate that RcGTA *g1* is the first gene of the RcGTA cluster and *g16* is likely to be the last. To assess the function of *g16* and *cysE1*, individual gene knockouts were produced by GTA-mediated gene replacement.

### There are two functional *cysE* genes in R. capsulatus.

**FIG 2:**
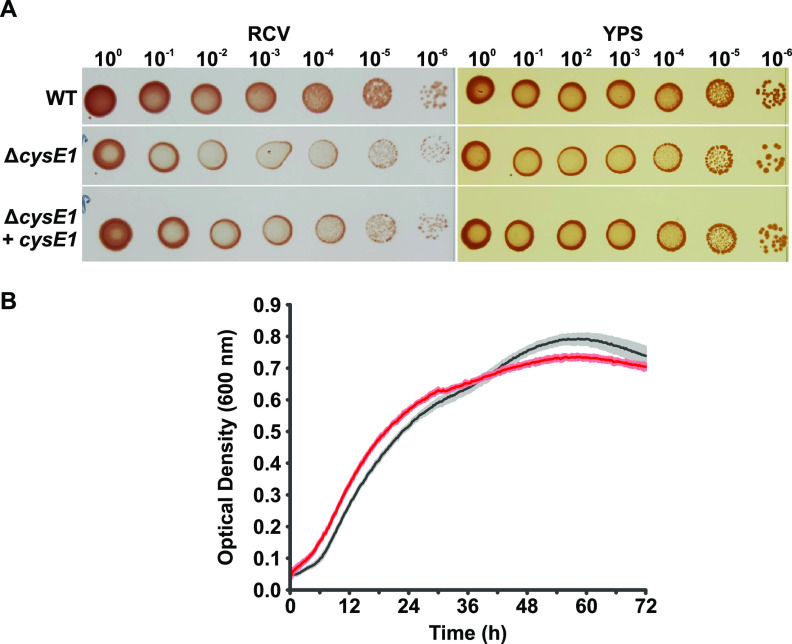
Deletion of the R. capsulatus *cysE1* gene does not lead to auxotrophy. (A) Serial dilutions of cultures of R. capsulatus SB1003 (WT), an isogenic *cysE1* knockout containing empty pCM66T plasmid (Δ*cysE1*), and the *cysE1* knockout complemented with pCM66T-*cysE1* (Δ*cysE1 *+* cysE1*) were spotted onto either minimal-medium (RCV) or complex-medium agar plates (YPS). (B) Optical density growth curve of R. capsulatus SB1003 (black line; gray shading shows the standard deviation) and an isogenic *cysE1* knockout (red line; pink shading shows the standard deviation) (*n* = 10). Cultures were grown in 200 μL of RCV liquid growth medium, and optical density at 600 nm was measured at 15-min intervals for 72 h.

The cysteine biosynthesis pathway is an essential biological process, but the R. capsulatus Δ*cysE1* strains were not auxotrophic when grown on RCV minimal medium (Fig. 2). This unexpected viability can be explained by the presence of an additional serine *O*-acetyltransferase gene elsewhere in the genome, *cysE2* (*rcc02246*; protein accession no. ADE85976). ClustalΩ alignment of the two *cysE* genes revealed 51.62% DNA sequence identity, and the protein products are 29.57% identical.

Previous transcriptome data showed that *cysE2* is expressed at lower levels than *cysE1* in wild-type R. capsulatus SB1003, and this was confirmed here by qPCR; *cysE2* transcripts were ~10-fold less abundant than *cysE1* in wild-type cells (Fig. S1A). Interestingly, *cysE2* transcript abundance increased by 2- to 3-fold in *cysE1* null mutants, albeit with a final concentration that was still only ~25% of *cysE1* transcript abundance in wild-type (WT) cells (Fig. S1A and B). Therefore, it is possible that limited upregulation of *cysE2* expression can partially mitigate the loss of *cysE1*. Similar to the Δ*cysE1* strain, deletion of *cysE2* did not lead to an auxotrophic phenotype (Fig. S1C and D). A double *cysE1 cysE2* mutant could not be made even using complex YPS medium, suggesting that loss of all serine *O*-acetyltransferase activity has wider implications than simply production of amino acids. In contrast to *cysE1*, *cysE2* is not upregulated in the RcGTA overproducer DE442 compared to wild-type SB1003 (23, 44).

It is worth noting that growth of the Δ*cysE1* and Δ*cysE2* strains appears paler than the wild-type on solid media, and both produce slightly smaller colonies (Fig. 2A and Fig. S1C and D). This is particularly evident on minimal medium. The reason for this phenotype is unclear, but the number of colonies is equivalent to that of the wild type (Fig. 2A and Fig. S1C and D), indicating that the cultures reached the same cell density. The plates were left for several more days to see if the morphology of the *cysE* strains changed after longer incubation, as would be expected if they were simply growing more slowly. No substantial changes were observed with longer incubation times. The rate of logarithmic growth in liquid minimal medium was comparable between the wild-type and Δ*cysE1* strains, but there was some divergence at the transition to stationary phase (Fig. 2B). Both growth curves began to plateau and then rose again shortly after, with the rise being more prominent for the wild-type (Fig. 2B). The numbers of CFU for both strains at stationary phase were comparable (Fig. 2A); therefore, it is unlikely that the increase in optical density was due to growth.

**FIG 3:**
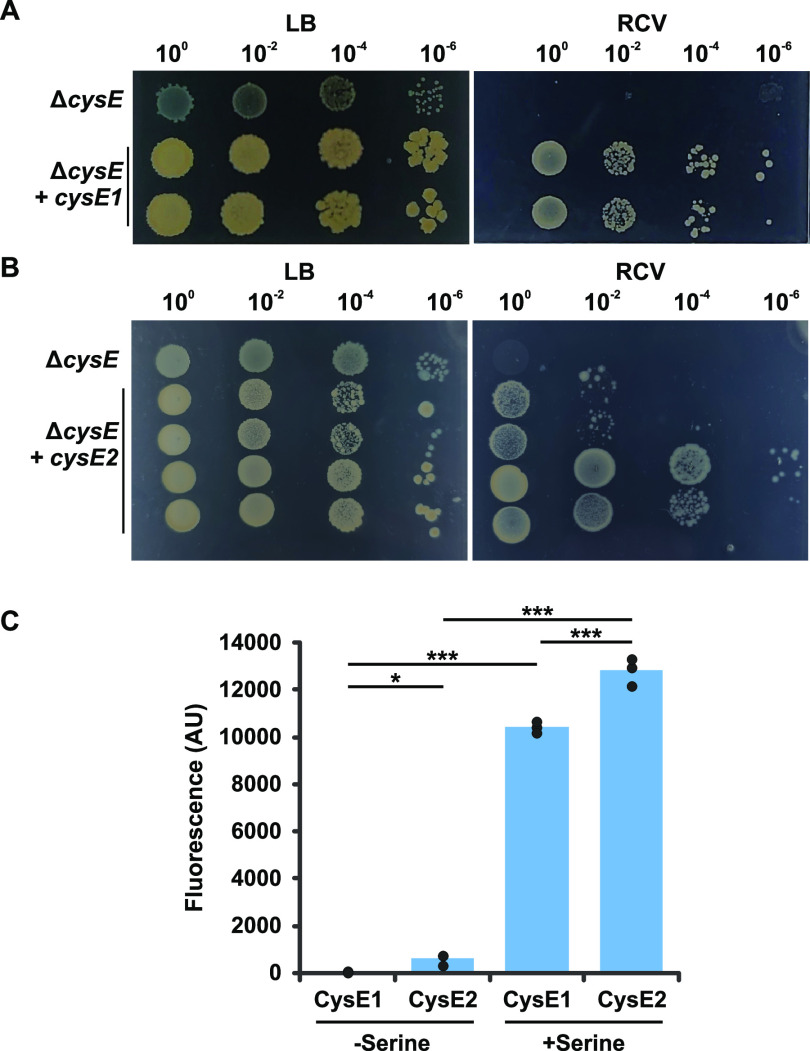
The R. capsulatus CysE1 and CysE2 proteins are both functionally active. (A and B) Serial dilutions of cultures of an E. coli K-12 strain BW25113 *cysE* knockout containing empty pCM66T plasmid (Δ*cysE*), the *cysE* knockout complemented with pCM66T-R. capsulatus *cysE1* (Δ*cysE* + *cysE1*), and the *cysE* knockout complemented with pCM66T-R. capsulatus *cysE2* (Δ*cysE* + *cysE2*) were spotted onto either minimal-medium (RCV) or complex-medium agar plates (LB). (C) Purified R. capsulatus CysE1 and CysE2 proteins were tested for *in vitro* transfer of CoA from acetyl-CoA to serine in a fluorescence-based assay. Reactions were carried out in the presence or absence of the serine receiver (−Serine/+Serine). Bars represent means, and filled circles are the individual data points. Statistical significance is indicated above the chart (one-way analysis of variance [ANOVA] using the Holm-Šidák test; *, *P* < 0.05; ***, *P* < 0.001).

The CysE1 and CysE2 proteins contain most of the conserved amino acids that are predicted to be involved in catalytic activity (Fig. S2). To test whether both proteins are functional, plasmids containing the R. capsulatus *cysE1* or *cysE2* gene were transformed into an auxotrophic E. coli Δ*cysE* mutant from the Keio collection (56). As expected, the E. coli Δ*cysE* strain could grow on LB agar plates but no growth was observed on RCV minimal agar. The ability to grow on RCV was restored by in *trans* complementation with the R. capsulatus *cysE1* or *cysE2* gene (Fig. 3A and B). The R. capsulatus CysE1 and CysE2 proteins were also purified to homogeneity by affinity chromatography for use in an *in vitro* serine acetyltransferase assay. Both proteins possessed clear enzymatic activity (Fig. 3C). CysE2 also had low-level activity in the absence of a serine acceptor and slightly higher overall activity in the presence of serine, possibly indicating autoacetylation of one of the CysE2 serine residues or the presence of an intermediate state (Fig. 3C).

To our knowledge, the presence of multiple *cysE* gene copies in bacteria has been reported only for *Lactobacillus* (57, 58), while redundant serine acetyltransferase activity was shown in Rhizobium leguminosarum but the gene responsible was not identified (59). Sequence homology searches using BLASTp revealed that CysE1 has long been associated with the *Rhodobacterales* (196 hits out of 201 [Table S2]; database update, 16 May 2022), whereas CysE2 is likely to have been recently acquired by horizontal gene transfer: only 26 hits out of 186 against *Rhodobacterales* but 148 hits against betaproteobacterial species (Table S3) (database update, 16 May 2022). The GC content of the *cysE1* gene is 64.7%, which is slightly closer to the genome average (66.6%) than that of *cysE2* (70.0%). Furthermore, CysE2 is predicted to be part of a KEGG pathway with gene products encoded on the endogenous R. capsulatus plasmid pRCB133 rather than by chromosomal genes (44). A homologue of CysE2 was also identified in R. leguminosarum (Fig. S2), which is likely to be the source of the residual serine acetyltransferase activity observed by Parker et al. (59). Given the data presented above, it is probable that the two R. capsulatus *cysE* genes have a redundant function with regard to essential biosynthesis.

### CysE1 affects the production of cell-associated EPS.

**FIG 4:**
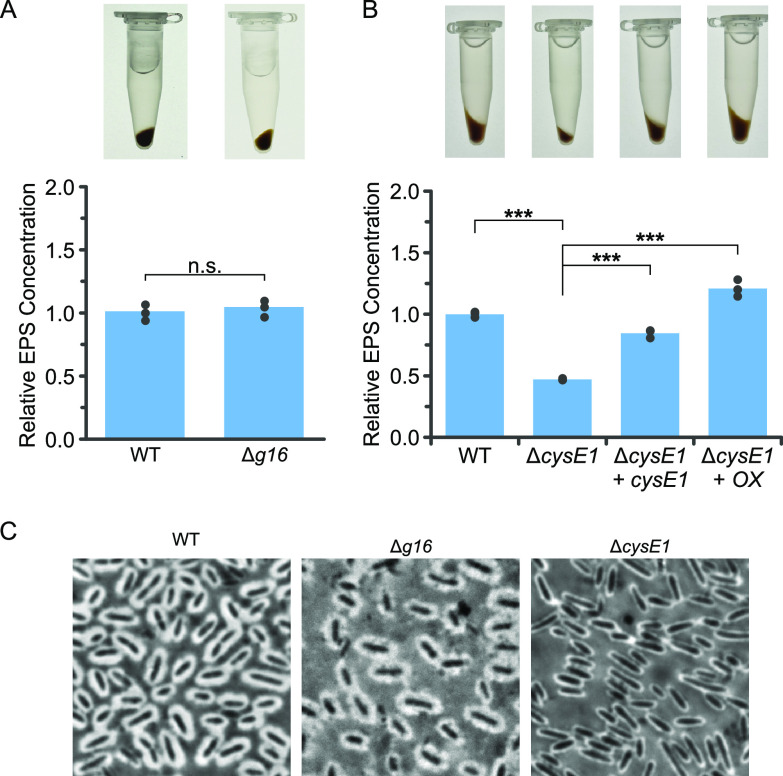
EPS and capsule production by R. capsulatus. (A and B) Quantification of cell-associated extracellular polysaccharide produced by the R. capsulatus derivatives annotated on the *x* axes, relative to the wild-type R. capsulatus parental strain. Images of the pellets formed after centrifugation of 1 mL of cells in the presence of 250 mM salt are provided above the respective bars. Bars represent means, and filled circles are the individual data points. Statistical significance is indicated above each chart (one-way ANOVA using the Holm-Šidák test [*n* = 3]; n.s., no significance; ***, *P* < 0.001). (C) Negative stain of the R. capsulatus capsule. Cells and background are stained dark with crystal violet, while the capsule itself is counterstained with copper sulfate and presents as a light halo around the cells. All images were taken using a 100× objective.

Visual inspection of cell pellets produced by *cysE1* mutants revealed a decrease in the characteristic loose mucoidy phenotype of the wild-type, whereas deletion of *g16* had no obvious effect (Fig. 4A and B). Loose-pellet formation has previously been attributed to accumulation of cell-associated extracellular polysaccharide (EPS) (13). The amount of EPS in the wild-type and mutant strains was quantified by EDTA extraction and phenol-sulfuric acid colorimetric quantification (13). This method does not distinguish between capsular EPS and lipopolysaccharides (LPS); however, LPS is not thought to be involved in the mucoidy pellet formation or with any RcGTA phenotypes (13). For simplicity, the extracted polysaccharides are referred to as EPS here. In agreement with the visual inspection, deletion of *g16* had no effect on EPS production (Fig. 4A), but deletion of the *cysE1* gene reduced EPS secretion to 47.0% of that of the wild type (Fig. 4B). Complementation in *trans* with a plasmid containing the *cysE1* gene expressed from its native promoter partially restored cell-associated EPS production to 84.6% of that of the wild type, and expression from the *puf* photosynthesis promoter (see Materials and Methods) overrescued production to 120.8% (Fig. 4B). We hypothesize that the increase in optical density (OD) in early stationary phase shown in Fig. 2B was a result of EPS production and that the smaller increase in OD for the *cysE1* knockout (KO) could reflect the impaired EPS production phenotype (Fig. 2B and 4). Deletion of *cysE2* had a lesser effect on EPS production, ≥78.8% of the wild-type level (Fig. S4A).

To determine whether the reduction in EPS was also associated with visibly impaired capsule formation, cells were negatively stained using crystal violet and copper sulfate (60). Consistent with the EPS data, substantial thinning of the capsule was observed for Δ*cysE1* cells but not Δ*g16* cells (Fig. 4C). The correlation between decreased EPS production and impaired capsule formation is also supported by a previous study of a R. capsulatus polysaccharide biosynthesis cluster (13). Overexpression of either *g16* or *cysE1* also significantly increased the levels of EPS produced to 136.7% (*P* < 0.001) and 170.0% (*P* < 0.001), respectively, of the wild-type level (Fig. S5). The precise role of *g16* is not clear and requires further investigation, but it does appear to directly or indirectly affect EPS production when expression levels are elevated. The connection between *g16* and *cysE1* is also supported by several examples in sequenced genomes where the two genes are fused (Fig. S6).

### Effect of *O*-acetyl-l-serine on wild-type R. capsulatus B10 cells.

**FIG 5:**
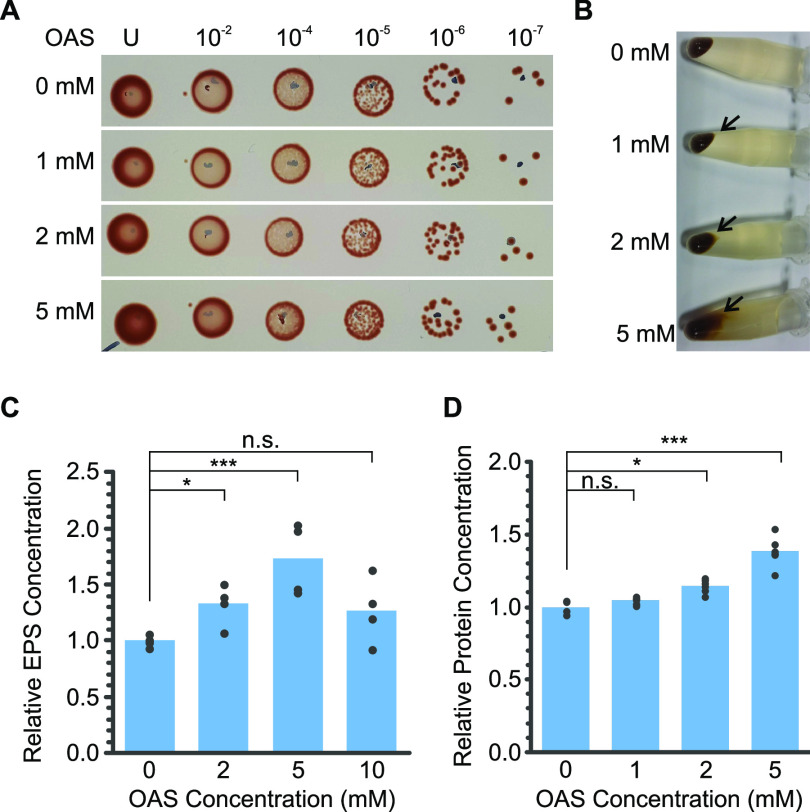
Effect of OAS on EPS and protein production by wild-type R. capsulatus SB1003. In all panels, data were obtained from wild-type R. capsulatus cultures with or without OAS treatment (0, 1, 2, 5, or 10 mM, as indicated). (A) Ten-microliter spot dilution series to assess the effect of OAS on CFU. Dilution factors are given across the top. U, undiluted. (B) Cell pellets from 1 mL of culture show increased mucoidy appearance in the presence of OAS. NaCl (250 mM) was added prior to centrifugation to facilitate pellet formation. The black arrow highlights loose-pellet formation. (C) Quantification of cell-associated extracellular polysaccharide produced by wild-type cells in response to the concentrations of OAS annotated on the *x* axes (*n* = 5). (D) Quantification of cell-associated extracellular protein produced by wild-type cells in response to the concentrations of OAS annotated on the *x* axes (*n* = 6). Bars represent means (*n* = 4), and filled circles are the individual data points. Statistical significance is indicated above each chart (one-way ANOVA using the Holm-Šidák test; n.s., no significance; *, *P* < 0.05; ***, *P* < 0.001).

**FIG 6:**
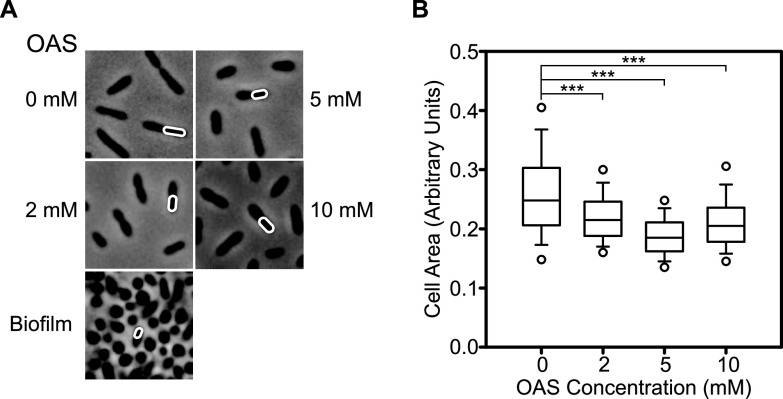
Effects of AOS on cell morphology. (A) Phase-contrast microscopy of wild-type R. capsulatus SB1003 cells at ×100 magnification. The R. capsulatus cells are rod shaped when grown in RCV defined medium but truncate in the presence of OAS. *Rhodobacter* are usually found in chains of two cells; individual exemplar cells are highlighted with a white outline. Images of typical biofilm cells are shown for comparison. (B) Box plot of cell size (area) observed after OAS treatments. Measurements were made using ImageJ from a total of five fields of view from two independent cultures per condition. The ends of the boxes define the 25th and 75th percentiles, with a line at the median. Error bars define the 10th and 90th percentiles, and white circles define 5th and 95th percentiles. Statistical significance is indicated above the plots (one-way ANOVA using the Holm-Šidák test [0 mM, *n* = 4,488; 2 mM, *n* = 7,947; 5 mM, *n* = 6,115; 10 mM, *n* = 9,363]; ***, *P* < 0.001).

The CysE1 enzyme produces *O*-acetyl-l-serine (OAS) from acetyl-CoA and serine (48, 61). To test the effect of OAS on wild-type R. capsulatus cells, various concentrations were added directly to late-log-phase cultures (Fig. 5 and 6). OAS had no effect on the number of CFU (Fig. 5A) but did increase loose-pellet formation (Fig. 5B), cell-associated EPS (Fig. 5C), and protein accumulation (Fig. 5D). At 5 mM OAS, EPS quantities peaked at 172.8% compared to the buffer-only control (Fig. 5C). Meanwhile, extracellular protein concentration rose to 138.4% with 5 mM OAS (Fig. 5D). Interestingly, OAS also altered cell morphology from typical rods to truncated rods/cocci, reminiscent of the biofilm-type morphology (Fig. 6A). Quantification of the area of individual cells indicated that the median area decreased to 74.6% in the presence of 5 mM OAS compared to 0 mM (Fig. 6B). Taken together, the data presented here corroborate the hypothesis that deletion of *cysE1* and subsequent reduction of OAS biosynthesis lead to the various phenotypes observed.

### CysE1 is required for optimal RcGTA receipt.

**FIG 7:**
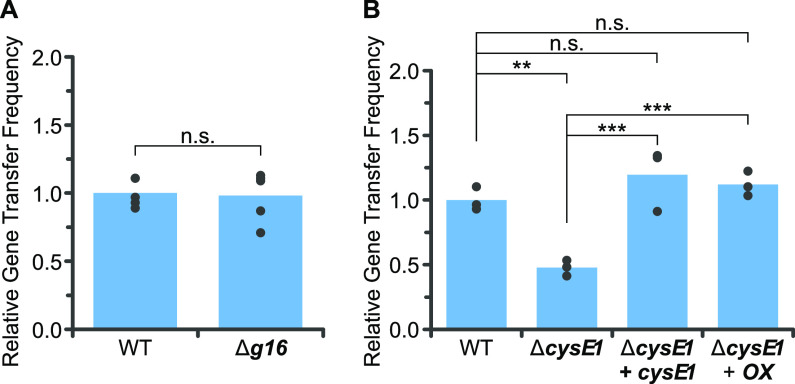
Effect of *g16* and *cysE1* genes on RcGTA receipt capability. Representative bar charts of the frequencies of rifampicin resistance transfer between R. capsulatus DE442 and sensitive recipient strains in a GTA bioassay. All gene transfer frequencies were normalized to wild-type R. capsulatus B10 (WT). (A) The WT recipient (*n* = 5) was compared to an isogenic RcGTA *g16* knockout strain (Δ*g16*, *n* = 5). (B) The WT recipient (*n* = 3) was compared to an isogenic *cysE1* knockout (Δ*cysE1*, *n* = 3). The *cysE1* knockout was complemented in *trans* with either *cysE1* expressed from its native promoter (+ *cysE1*; *n* = 3) or from the *puf* photosynthesis promoter (+ OX; *n* = 3). All other wild-type and mutant strains carried the empty pCM66T plasmid. In each case, bars represent means and filled circles are the individual data points. Statistical significance is indicated above the chart (one-way ANOVA using the Holm-Šidák test; n.s., no significance; **, *P* < 0.01; ***, *P* < 0.001).

It is known that EPS is required for production of the R. capsulatus capsule, which is in turn required for optimal RcGTA infection (13, 15). Because expression levels of *g16* and *cysE1* affect EPS production/capsule formation and transcription of both genes is coregulated with the RcGTA genes (Fig. 1), we hypothesized that there may also be an effect on RcGTA receipt. Standard GTA bioassays were carried out to monitor the transfer of antibiotic resistance from a rifampicin-resistant donor to a rifampicin-sensitive recipient (R. capsulatus B10). Wild-type strains of R. capsulatus produce RcGTAs at low frequency; therefore, the RcGTA overproducer strain DE442 was used as a donor in all assays to increase sensitivity (22, 37, 62). We found that deletion of *g16* did not significantly decrease receipt of RcGTA DNA compared to that in wild-type B10 (98.2%, *P* = 0.837). The frequency of RcGTA receipt by the Δ*cysE1* derivative was 47.7% compared to the control (Fig. 7). Complementation of the B10 Δ*cysE1* mutant with a plasmid containing the *cysE1* gene expressed from its native promoter restored RcGTA recipient efficiency to 119.5% of the wild type value, and expression from the *puf* promoter rescued it to 112.1% (Fig. 7B). Meanwhile, deletion of *cysE2* had no significant effect on RcGTA receipt (Fig. S4B). Overall, these data indicate that CysE1 is required for optimal receipt of RcGTA DNA.

### Biofilm production is impaired in a *cysE1* deletion strain.

**FIG 8:**
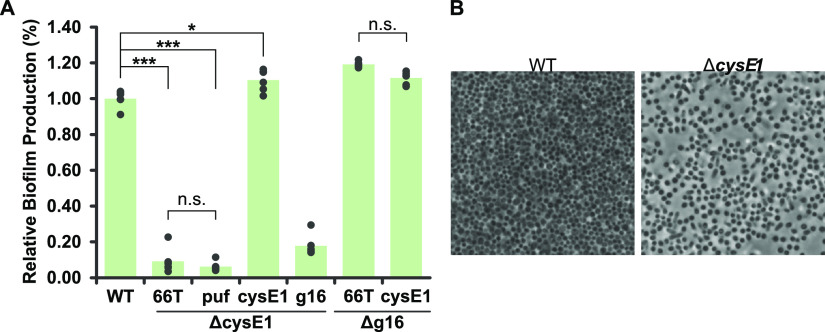
Effect of *cysE1* deletion on biofilm. (A) Quantification of relative biofilm production on an uncoated polystyrene microtiter plate surface. Wild-type R. capsulatus SB1003 (WT) was compared to isogenic *cysE1* (Δ*cysE1*) and g16 (Δg16) knockouts. Knockout strains were complemented in *trans* with empty pCM66T plasmid (66T) or pCM66T containing the *puf* photosynthesis promoter alone (puf), *puf-cysE1* (cysE1), or *puf*-*g16* (g16). Bars represent means (*n* = 6), and filled circles are the individual data points. Statistical significance is indicated above the chart (one-way ANOVA using the Holm-Šidák test; n.s., no significance; *, *P* < 0.05; ***, *P* < 0.001). (B) Representative phase-contrast images (×100 magnification) of R. capsulatus biofilm formed on an uncoated glass slide.

EPS is also known to be a key prerequisite for bacterial adherence to solid surfaces and formation of three-dimensional biofilm architecture (6, 63), so we tested the ability of the Δ*cysE1* strain to form biofilms on untreated polystyrene and glass surfaces. To assess biofilm production, cultures were incubated statically for 5 days in 96-well flat bottom polystyrene microtiter plates. Quantification of the adhered biomass agreed with the biochemical and histochemical assays for EPS/capsule. Biofilm production by the SB1003 Δ*cysE1* mutant was 9% of that of the wild type (Fig. 8A). Complementation of the Δ*cysE1* strain in *trans* restored biofilm production to 110% (Fig. 8A). Comparable complementation with the *puf* promoter alone or *puf-g16* did not restore biofilm formation. Furthermore, deletion of the *g16* gene did not decrease biofilm formation, and complementation of SB1003 Δ*g16* with *puf-cysE1* did not further increase biofilm production beyond wild-type levels (Fig. 8A). Meanwhile, biofilms were allowed to form for 5 days on glass microscope coverslips and then imaged by phase-contrast microscopy. Wild-type R. capsulatus cultures readily produced evenly distributed, densely packed biofilms across the full surface of the coverslip (Fig. 8B). Although Δ*cysE1* strains were capable of forming biofilm, the overall cell density was substantially lower than that of the wild type, with fewer close cell-cell contacts and frequent gaps visible throughout the colonized surface (Fig. 8B). Biofilms produced by Δ*g16* strains were indistinguishable from those of the wild type. Overall, the data presented clearly indicate that *cysE1* is involved in regulation of extracellular polymeric substances and biofilm production.

## DISCUSSION

In this study, we investigated the roles of two genes, *g16* and *cysE1*, immediately downstream of the R. capsulatus core GTA gene cluster. The impact of the CysE1 serine *O*-acetyltransferase on RcGTA receipt, secretion of polymeric substances to the cell surface, and formation of biofilm was clearly observed, but the role of RcGTA *g16* is more enigmatic. Deletion of the *g16* gene had no significant impact on any of the phenotypes tested in this study (production of EPS, biofilm and capsule formation, or RcGTA activity). However, overexpression did lead to an increase in EPS production. Attempts to identify a function for the g16 protein via sequence and structural homology searches yielded no high-confidence hits. Clearly, further work is required to definitively identify the role RcGTA g16 plays and the nature of its possible interaction with CysE1.

GTA production is known to be influenced by external nutrient availability (31, 64), and Westbye et al. (31) showed that chemical inhibition of histidine biosynthesis boosted RcGTA production. The data presented here are the first to link an amino acid biosynthesis pathway to regulation of RcGTA recipient ability. CysE catalyzes the production of OAS and its spontaneous racemer NAS (Fig. S7) (50, 65). In addition to being precursors to cysteine, OAS and NAS are also signaling molecules. NAS is a ligand for CysB, a LysR family transcriptional regulator that autoinhibits its own expression and activates expression of various sulfur metabolism and amino acid biosynthesis regulons (50, 51, 66, 67). CysB is not currently annotated in the R. capsulatus SB1003 genome, but there are at least 20 putative LysR-type regulators, including *rcc02272*, which is a strong HHPRED match to Klebsiella aerogenes CysB (PDB code 1AL3; E value, 1.3e−46) (Table S4).

Previous studies in other species have determined that CysE is involved in biofilm formation. The mechanism, however, is not fully understood, and there is some variation from species to species. Escherichia coli and Providencia stuartii *cysE* is required for production of an unidentified extracellular signal that promotes differential gene expression, and deletion of the *cysE* gene leads to increased biofilm formation (50). Addition of OAS but not NAS complemented the null phenotype, and this response was *cysB* dependent (50). In contrast, Vibrio fischeri *cysE* is an essential gene, and CysE is required for synthesis of an extracellular signal that promotes biofilm formation (68). It is also notable that a recent study identified two natural products as inhibitors of Staphylococcus aureus CysE, both of which also inhibited biofilm formation and even promoted dissolution of mature biofilm (69).

CysB is also known to regulate expression of diverse genes, such as those encoding acid tolerance (70, 71), antibiotic resistance (72, 73), type III secretion (74), and 4-hydroxy-2-alkylquinolones (HAQs). HAQs are core constituents of Pseudomonas aeruginosa cell-to-cell signaling that control various physiological functions, such as the secretion of extracellular DNA and biofilm formation (73, 75). PqsR is the direct regulator of HAQ synthesis, and *pqsR* expression is in turn regulated by CysB via a cysteine-independent mechanism (73). A similar mechanism could explain the phenotypes observed here, i.e., a reduction of OAS/NAS production in R. capsulatus could lead to impaired quorum sensing, which is known to be an important regulator of both EPS biosynthesis and RcGTA production/receipt (13, 14).

Under normal laboratory conditions, GTA genes are expressed in only a small subset of a given bacterial population; for example, ~10% of Bartonella henselae cells spontaneously produce GTAs (64), as do ~1 to 3% of R. capsulatus cells (22, 25). Moreover, wild-type strains produce modest *in vitro* transduction frequencies of no more than 10^−4^ (64, 76, 77). In contrast, the only large-scale *in situ* study to date reported frequencies of gene transfer that were orders of magnitude higher (43). Some of the discrepancy between the *in vitro* and *in situ* data could in part be due to poor replication of the optimal natural conditions for GTA transfer in the lab. Indeed, simply coculturing marine GTA producers from the *Roseobacter* clade with a natural symbiont, *Synechococcus*, led GTA proteins to constitute up to 13% of total extracellular protein (78). Mathematical modeling has raised doubts about whether GTA gene transfer, at the frequencies reported *in vitro*, would provide sufficient advantage to producer cells via recombination/allelic diversity alone, to outweigh the cost of lysis (41). Some of these reservations could be allayed by increased production of GTAs in a setting where potential recipients are at high density, receptor polysaccharide is enhanced, and loss of GTAs by diffusion is more constrained (Fig. S8).

We propose that an environmental niche for R. capsulatus GTA production could be in biofilm and that RcGTA activity is coregulated with biofilm production, although more work will be required to definitively prove this. We have shown that the two genes immediately adjacent to the known RcGTA cluster are coregulated with the RcGTA genes and one, *g16*, is also cotranscribed. Deletion of *cysE1* leads to a substantial decrease in extracellular polysaccharide and substantial thinning of the capsule; EPS and capsule are both essential for successful adsorption to and infection of a target cell by RcGTAs (13–15). In a variety of species, polysaccharide secretion in particular is known to be a prerequisite for optimal biofilm formation (6, 79, 80), and here, we also observed substantial loss of biofilm formation by the R. capsulatus *cysE1* knockout. This study focused on R. capsulatus, but GTAs are found in many species (17, 38), most of which are known to form biofilms, and therefore, our hypotheses could be tested in other species in the future.

We hypothesize that CysE1 is a constituent of interlocking biofilm and RcGTA regulatory circuits. For example, FliQ and CheY are known contributors to biofilm, and both are under the control of CtrA, a well-characterized pleiotropic regulator that is essential for RcGTA production (81–83). Furthermore, a recent global expression analysis of an *Agrobacterium pdhS2* mutant identified a regulon that included control of motility, production of adhesive polysaccharides, surface attachment, and biofilm production via c-di-GMP signaling (84). Interestingly, homologues of most of the core components of the *Agrobacterium* biofilm regulation pathway (CtrA, CckA, PleC, and DivK) and c-di-GMP are all established regulators of RcGTA production (23, 32, 33, 35, 82, 85).

It is generally accepted that high levels of c-di-GMP are associated with reduced motility and increased biofilm formation in multiple species (75). For example, c-di-GMP levels in biofilm cells from the alphaproteobacterium Ruegeria mobilis are 20-fold higher than in planktonic cells (86). In contrast, there is only limited information about the role of c-di-GMP in GTA regulation. Two recent papers reported that increased c-di-GMP concentrations repress both RcGTA production and flagellar motility in R. capsulatus (33, 34); however, both high and low c-di-GMP concentrations appear to be important for different stages of RcGTA production and release (87). It is likely that multiple factors, such as the precise spatiotemporal levels of c-di-GMP, phosphorylation states of essential regulators (e.g., CtrA or CckA), and input from other signaling molecules (e.g., homoserine lactone [HSL], rcc00280, OAS/NAS, etc.), all combine to provide fine control of RcGTA expression and biofilm formation (14, 32, 35–37, 82, 87). More work is needed to establish the complex regulatory mechanisms, but our data provide the first link between regulation of GTA gene transfer and production of biofilm. Establishing the environmental niche for GTAs is essential to fully understand their impact on host communities and to effectively identify new GTAs in a broader range of species.

## MATERIALS AND METHODS

### Bacterial strains and growth conditions.

Three Rhodobacter capsulatus strains were used in this study: rifampicin-sensitive wild-type strain B10 (88), the rifampicin-resistant derivative SB1003 (ATCC BAA-309), and the RcGTA overproducer strain DE442 (23, 37, 62). All R. capsulatus cultures were grown at 30°C either with aeration in the dark or in anoxic sealed tubes under constant illumination. Two growth media were used, YPS complex medium (0.3% [wt/vol] yeast extract, 0.3% [wt/vol] peptone, 2 mM MgCl_2_, 2 mM CaCl_2_) and RCV defined medium (89); 1.5% (wt/vol) agar was added for solid media. E. coli strain S17-1, which contains chromosomally integrated *tra* genes, was used as a donor for all conjugations. NEB 10-beta competent E. coli (New England Biolabs [NEB], Ipswich, MA, USA) were used for standard cloning and plasmid maintenance. E. coli K-12 BW25113 Δ*cysE* was sourced from the Keio collection (56). All E. coli cultures were grown at 37°C in LB medium. Antibiotics were used for selection and counterselection as follows: for R. capsulatus, rifampicin at 100 μg/mL, gentamicin at 3 μg/mL, and kanamycin at 10 μg/mL; for E. coli, gentamicin at 10 μg/mL and kanamycin at 50 μg/mL. OAS (1 M solution in 10 mM Tris, pH 6; Sigma-Aldrich, Poole, UK) was added directly to cultures to the indicated final concentrations.

### General cloning.

All cloning reactions were carried out with the In-Fusion cloning kit (TaKaRa Bio Europe, Saint-Germain-en-Laye, France) or NEBuilder (NEB) to produce the constructs listed in Table 1. Inserts were amplified using primers with 15-bp 5′ overhangs of complementary sequence to the DNA with which it was to be joined. Oligonucleotides were obtained from either Integrated DNA Technologies (IDT) or Sigma (Table 2). Q5 Polymerase (NEB) was used for all amplifications. pCM66T was used as a broad-host-range destination vector for all constructs and was linearized with BamHI (NEB).

**TABLE 1:** Plasmids used in this study^*a*^

Name	Description	Vector	Source or reference
pCM66T	Broad-host-range vector (gift from Mary Lidstrom); p*tac* IncP ColE1	NA	Addgene plasmid 74738
pETTF11	Protein expression plasmid, His6-3c tag, T7 inducible promoter	NA	94
pmCRc	Codon-optimized mCherry	pEX	Eurofins Genomics
pCMF130	RcGTA promoter fused to codon-optimized mCherry	pCM66T	This study
pCMF151	*puf* promoter fused to SB1003 RcGTA *g16* gene	pCM66T	This study
pCMF152	*puf* promoter fused to SB1003 *cysE1* gene	pCM66T	This study
pCMF278	puf promoter only control	pCM66T	This study
pDS1	*cysE1* gene and flanking DNA	pCM66T	This study
pDS2	*g16* gene and flanking DNA	pCM66T	This study
pDS3	cysE1::Gent	pCM66T	This study
pDS4	g16::Gent	pCM66T	This study
pDS5	g16-cysE1::Gent	pCM66T	This study
pDS6	*cysE2* gene and flanking DNA	pCM66T	This study
pDS7	*cysE2*::Gent	pCM66T	This study
pDS8	*cysE2*::Kan	pCM66T	This study
pCMF140	His6-CysE1	pETTF11	This study
pDS9	His6-CysE2	pETTF11	This study

a NA, not applicable.

**TABLE 2:** Oligonucleotides used in this study

Use and name	Sequence (5′-3′)	Construct produced^*a*^
RT-PCR		
yceG RT1 F	GGGATGCGGCTGCAGACCGATCC	NA
g1 RT1 R	GTCAACCTCCTGCGGCGTC	NA
g15-16 RT2 F	GGGAGGTGTTTCAATTCGCC	NA
g15-16 RT2 R	GGTAAAGATCGGCCCAATGCG	NA
g16-cysE1 RT3 F	GCTGGCACAATGGTTGC	NA
g16-cysE1 RT3 R	GTGAATATTGCCCAGCA	NA
cyse1-1701 RT4 F	AGGACATGGCCTATTTCGTGC	NA
cyse1-1701 RT4 R	CATCACATGGTTCAGGTTCG	NA
qPCR		
cysE1 qF	ATGCTGCATTCGGTGACA	NA
cysE1 qR	TGAATATTGCCCAGCACCTT	NA
cysE2 qF	CCAGCTTGACCAGGATGTC	NA
cysE2 qR	CGATGCGATGGTGGATCA	NA
uvrD qF	CAGAAGGAACACACGGTCAA	NA
uvrD qR	AAAGTGTCAGGCGGAATCTC	NA
Gene knockout constructs		
Gent F	GATCCCCTGATTCCCTTTGT	pDS3, pDS4 and pDS5
Gent R	CTTGAACGAATTGTTAGG	pDS3, pDS4 and pDS5
cysE1 Up F	CGACTCTAGAGGATCCAGGTGTCGGATGTGTATGGC	pDS1 and pDS3
cysE1 Up R	AACAATTCGTTCAAGCGGGTTTTGGACATGGGACT	pDS1 and pDS3
cysE1 Down F	GGGAATCAGGGGATCCGGTCAACATGGATCAGCAG	pDS1 and pDS3
cysE1 Down R	CGGTACCCGGGGATCCCAAAAACAGCCCGATCACCC	pDS1, pDS3, and pDS5
cysE2 Up F	CGACTCTAGAGGATCCGATGCGCTGGCCCAGATCC	pDS6, pDS7, and pDS8
cysE2 Up R-Gent	GGGAATCAGGGGATCACCGGAACCAGGGAAAACC	pDS7
cysE2 Down F-Gent	AACAATTCGTTCAAGATGTCTGGCTGACCGAAAGC	pDS7
cysE2 Down R	CGGTACCCGGGGATCTCGTCGTAATCGACGAAGG	pDS6, pDS7, and pDS8
cysE2 Up R-Kan	GACACATGCAGCTCCACCGGAACCAGGGAAAACC	pDS8
cysE2 Down F-Kan	ATTTTGAGACACAACATGTCTGGCTGACCGAAAGC	pDS8
g16 Up F	CGACTCTAGAGGATCCGGGAGGTGTTTCAATTCGCC	pDS2, pDS4, and pDS5
g16 Up R	AACAATTCGTTCAAGGTAAAGATCGGCCCAATGCG	pDS2 and pDS4
g16 Down F	GGGAATCAGGGGATCCGACGATCTTGCAGAGGTGA	pDS2 and pDS4
g16 Down R	CGGTACCCGGGGATCCCATCACATGGTTCAGGTTCG	pDS2 and pDS4
Overexpression constructs		
pPuf F	CGACTCTAGAGGATCCGAGCTTCGGAATCTGCG	pCMF151 and pCMF152
pPuf R	CATAACAACCTCCGGATTGGCAGAC	pCMF151 and pCMF152
cysE1 OX F	CCGGAGGTTGTTATGTCCAAAACCCGTGCG	pCMF152
cysE1 OX R	CGGTACCCGGGGATCGTCAGCCCTCGCAGTCGC	pCMF152
g16 OX F	CCGGAGGTTGTTATGCGGCCGATCCTGATC	pCMF151
g16 OX R	CGGTACCCGGGGATCTCACAGATCCGCATCCAC	pCMF151
puf con F	CGACTCTAGAGGATCCGAGCTTCGGAATCTGCG	pCMF278
puf con R	CGGTACCCGGGGATCCATAACAACCTCCGGATT	pCMF278
Reporter construct		
pGTA F	CGACTCTAGAGGATCGATTGTCGATCAGATCAC	pCMF130
pGTA R	GCTGACCATCGCCAGGGCCAGTTCC	pCMF130
mCherryRc F	CCTGGCGATGGTCAGCAAGGGGGAG	pCMF130
mCherryRc R	CGGTACCCGGGGATCCTGTTGTGTGGAATTGTGAGC	pCMF130
Protein purification		
H6-Cyse1 F	TCCAGGGACCAGCAATGATGTCCAAAACCCGTGCG	pCMF140
H6-CysE1 R	TGAGGAGAAGGCGCGTCAGCCCTCGCAGTCGCAGC	pCMF140
H6-Cyse2 F	TCCAGGGACCAGCAATGACGCTTGACACGCAACG	pDS9
H6-CysE2 R	TGAGGAGAAGGCGCGTCGGTCATTCACGTTCTCC	pDS9

a NA, not applicable.

### Transformation/conjugation.

E. coli was transformed with 100 ng of plasmid DNA by standard heat shock transformation (90). For conjugation, 1-mL aliquots of overnight cultures of the E. coli S17-1 donor and *Rhodobacter* recipient strains were centrifuged at 5,000 × *g* for 1 min, washed with 1 mL YPS, centrifuged again, and resuspended in 100 μL YPS. Ten-microliter portions of concentrated donor and recipient cells were mixed and spotted onto YPS agar or spotted individually as negative controls. Plates were incubated overnight at 30°C. Spots were scraped, suspended in 100 μL YPS broth, and plated on YPS containing 100 μg/mL rifampicin (counterselection against E. coli) and 10 μg/mL kanamycin (plasmid selection). Plates were incubated overnight at 30°C and then restreaked onto fresh agar to obtain pure single colonies.

### Gene knockouts.

Knockouts were created by GTA transfer. pCM66T plasmid constructs were created with a gentamicin resistance cassette flanked by 500 to 1,000 bp of DNA from either side of the target gene (Table 1). Assembly was achieved by a one-step, four-component NEBuilder (NEB) reaction and transformation into NEB 10-beta cells. Deletion constructs were introduced into the RcGTA overproducer strain, and a standard GTA transduction assay was carried out to replace the intact chromosomal gene with the deleted version.

### Microtiter plate growth curves.

Two hundred microliters of growth medium was dispensed into the wells of a 96-well microtiter plate. For each replicate, 4 μL of independently grown cells was seeded into the well. The plates were assayed in a SpectroStar Nano device (BMG Labtech, Ortenberg, Germany). Plates were held at a constant 30°C, and optical density readings were taken at 15-min intervals. Plates were agitated at 150 rpm using an orbital pattern for 1 min before every reading.

### Spot dilution assays.

Bacterial cultures were twice pelleted and resuspended in liquid minimal medium (RCV) to remove residual complex growth medium components or secondary metabolites. Washed cells were diluted in a 1-in-10 serial dilution series in RCV. Ten microliters from each dilution was spotted onto dry minimal-medium agar plates (RCV) or complex-medium agar plates (YPS or LB) and allowed to dry completely. Plates were incubated at 30°C for R. capsulatus or 37°C for E. coli.

### Gene overexpression.

Overexpression in *Rhodobacter* was achieved by a transcriptional fusion of the genes of interest to the *puf* photosynthesis promoter. The promoter contains 1,002 bp of sequence upstream of the *pufB* gene, with the entire *pufB* start codon directly preceded by each gene of interest. The promoter and its use for expression of heterologous genes were described previously (22, 91). Growth and general maintenance of *Rhodobacter* strains containing overexpression plasmids was carried out at 30°C under aerated, chemotrophic growth conditions, where transcription from the *puf* promoter is strongly repressed. To establish overexpression conditions, cultures were grown in sealed glass tubes and incubated at 30°C with illumination to induce *puf* promoter activity.

### *Rhodobacter* gene transfer assays.

*Rhodobacter* assays were carried out essentially as described in reference 92. RcGTA donor cultures were grown in YPS for ~72 h, and recipient cultures were grown in RCV for ~24 h. Cells were cleared from donor cultures by centrifugation, and the supernatant was filtered through a 0.45-μm syringe filter. Recipient cells were concentrated 3-fold by centrifugation at 5,000 × *g* and resuspension in 1/3 volume G buffer (10 mM Tris-HCl [pH 7.8], 1 mM MgCl_2,_ 1 mM CaCl_2,_ 1 mM NaCl, 0.5 mg/mL bovine serum albumin [BSA]). Reactions were carried out in polystyrene culture tubes (Starlab Ltd., Milton Keynes, UK) containing 400 μL G buffer, 100 μL recipient cells, and 100 μL filter donor supernatant, and then the mixtures were incubated at 30°C for 1 h. Nine hundred microliters of YPS was added to each tube and incubated for a further 3 h. Cells were harvested by centrifugation at 5,000 × *g* and plated on YPS containing 100 μg/mL rifampicin (for standard GTA assays) or 3 μg/mL gentamicin (for gene knockouts).

### Reverse transcriptase PCR (RT-PCR).

Total RNA was extracted from stationary growth phase R. capsulatus cultures using the NucleoSpin RNA minikit (Macherey-Nagel GmbH & Co., Düren, Germany), including on-column DNase treatment. Trace DNA contamination was eliminated by using a Turbo DNase-free kit (Thermo Fisher Scientific, Waltham, MA, USA). RNA was quantified with a Nanodrop instrument (Thermo Fisher Scientific), and cDNA was synthesized from 1 μg of total RNA using the LunaScrip RT SuperMix kit (NEB). PCR amplification across gene boundaries was carried out using 1 μL of cDNA, standard Q5 polymerase conditions, 25 cycles of amplification, and primers as listed in Table 2.

### Quantitative RT-PCR.

Dilutions (1:50) of the cDNA template were prepared in distilled water (dH_2_O), and 1 μL was used per reaction. Reactions contained Fast Sybr green Mastermix (Applied Biosystems, Waltham, MA), cDNA, and primers (500 nM). Standard conditions were used, with an annealing temperature of 60°C. All primer efficiencies were calculated as between 90 and 110%. Relative gene expression was determined using the ΔΔ*C_T_* method (93). For each sample, variance was calculated for three independent biological replicates, which were each the mean for three technical replicates. A QuantStudio 3 real-time PCR system was used for all experiments (Applied Biosystems).

### Protein purification.

For His6-tagged proteins, 500-mL cultures of E. coli containing the relevant expression plasmid were induced at mid-exponential growth phase with 0.2 mM IPTG (isopropyl-β-d-thiogalactopyranoside) overnight at 20°C (94). Concentrated cells were lysed in 20 mL binding buffer (0.5 M NaCl, 75 mM Tris; pH 7.75) plus 0.2 mg mL^−1^ lysozyme (Avantor-VWR, Radnor, PA) and 500 U Basemuncher endonuclease (Abcam, Cambridge, UK) for 30 min on ice and then sonicated. Cleared supernatant was applied to a 5-mL HisTrap FF crude column (Cytiva, Marlborough, MA), and the bound, His-tagged protein was eluted with 125 mM imidazole. Eluted protein was desalted on a HiPrep 26/10 desalting column (Cytiva) and then further separated by size exclusion chromatography on a HiLoad 16/60 Superdex 200 preparative-grade gel filtration column. All chromatography steps were carried out on an AKTA Prime instrument (Cytiva). Purified proteins were concentrated in a Spin-X UF centrifugal concentrator (Corning Inc., Corning, NY) and quantified by the extinction coefficient method and a NanoDrop 1000 Spectrophotometer (Thermo Scientific, Waltham, MA). Samples were stored at −80°C in binding buffer plus 50% glycerol.

### Transacetylation assay.

An endpoint assay for transacetylation was performed using a fluorometric acetyltransferase activity assay kit (Abcam) according to the manufacturer’s instructions. CysE1 and CysE2 proteins were used at a final concentration of 100 nM and l-serine at a final concentration of 0.25 mM. All reaction mixtures were incubated for 30 min at room temperature. Negative-control reactions were carried out in the absence of protein and/or l-serine. Fluorescence output was measured in an Infinite M200 microplate reader (Tecan, Männedorf, Switzerland) at excitation and emission wavelengths of 380 and 520 nm.

### Quantification of cell-associated extracellular substances.

Extracellular polysaccharide and protein were extracted from R. capsulatus cells grown in RCV medium under photosynthetic conditions to stationary growth phase. In brief, filtered NaCl solution was added to each culture to a final concentration of 250 mM, and 1-mL samples were harvested by centrifugation at 14,000 × *g* for 10 min. Addition of NaCl was found to improve the formation of tight cell pellets, which allowed the supernatant to be carefully removed and discarded. To remove residual free polysaccharides that may have been present in the supernatant, cell pellets were resuspended in 1 mL of 250 mM NaCl and harvested again by centrifugation. This wash step was sufficient to remove >90% of loosely associated or free polysaccharides (Fig. S3). Washed cell pellets were resuspended in 1 mL of 50 mM EDTA and incubated at 37°C for 3 h. The samples were cleared by centrifugation at 14,000 × *g* for 10 min, and the supernatant was carefully transferred to a fresh tube. EPS quantification was carried out essentially as previously described (13) using a modified phenol-sulfuric acid method and a calibration curve constructed using an equal-concentration mixture of sucrose and fructose. Each quantification reaction mixture consisted of 100 μL sample supernatant, 100 μL 5% (wt/vol) phenol, and 500 μL 93% (vol/vol) sulfuric acid. Tubes were incubated at room temperature for 10 min before the absorbance at 490 nm was measured in a spectrophotometer. Extracellular protein concentration was determined by bicinchoninic acid assay (BCA) and an albumin standard curve according to the manufacturer’s instructions (Abcam).

### Capsule staining.

Capsule formation was measured by negative staining (13, 60). In brief, 1-mL samples of stationary-phase R. capsulatus cultures were harvested by centrifugation, and the pellets were resuspended in 1 mL of 1% (wt/vol) Carnation skim milk powder. The mixture was smeared onto a microscope slide and allowed to air dry. The cells were stained with 1% (wt/vol) crystal violet for 5 min and then washed with 20% (wt/vol) copper sulfate. Slides were allowed to air dry before being imaged by oil immersion phase-contrast microscopy at ×100 magnification. Original images were black and white, and *post factum* false color was added using ImageJ to approximate the original image.

### Biofilm assays.

R. capsulatus cultures were grown in 200 μL YPS medium in uncoated, polystyrene 96-well plates. Plates were incubated at 30°C, in the dark and without agitation, for 5 days. The culture medium was decanted and the wells were washed 5 times with dH_2_O to remove any unadhered cells. The cells were stained with 0.1% (wt/vol) crystal violet for 10 min, washed 5 times with dH_2_O to remove excess dye, and allowed to air dry. Bound crystal violet was resolubilized with 250 μL of 30% (vol/vol) acetic acid, and absorbance was measured at 590 nm. For coverslip assays, R. capsulatus cells were inoculated into 10 mL of YPS medium in a 50-mL Falcon tube containing a partially submerged glass coverslip and incubated for 5 days statically at 30°C in the dark or with illumination, as indicated. The coverslip was removed from the tube and washed thoroughly in dH_2_O, and excess liquid was wicked away with blotting paper. The biofilm was then sandwiched between a microscope slide and a fresh coverslip. Imaging of the mature biofilm was carried out using an Axioscope inverted microscope (Carl Zeiss Ltd., Cambridge, UK) and mounted charge-coupled device (CCD) camera.

### Data analysis.

The RNA-seq data set in the NCBI Gene Expression Omnibus (GEO) database, accession number GSE118116, was analyzed to assess transcriptional units and compared to the meta-analysis of GSE18149, GSE33176, GSE41014, and GSE53636 carried out by Peña-Castillo et al. (44). Transcript abundance was visualized using the Broad Institute’s IGV viewer (95). Protein alignments were made in ClustalΩ (96) and then visualized in Jalview (97). FIJI software (98) was used to analyze microscopy images. Statistical analysis was carried out using SigmaPlot software version 13 (Systat Software Inc., Slough, UK; www.systatsoftware.com), and for each use, the test parameters are indicated in the text and/or figure legends. All data presented in figures correspond to one representative experiment consisting of at least three biological replicates; all experimental data were confirmed on at least two different occasions. CorelDraw 2018 (Corel Corporation, Ottawa, Canada) was used for figure preparation and ChemDraw (Perkin Elmer, Waltham, MA, USA) for chemical structure diagrams.

### Sequence similarity analysis.

NCBI BLASTp searches to identify homologues of R. capsulatus SB1003 proteins were performed using the default parameters: expect threshold = 0.05, word size = 6, blosum62 similarity matrix, gap costs of existence = 11, and extension = 1. Queries were made against the nonredundant protein sequences database (nr; posted 5 May 2022, accessed 16 May 2022). A summary of the full outputs for CysE1 and CysE2 can be found in Tables S2 and S3. HHPRED analysis of the R. capsulatus SB1003 *rcc02272* gene product was carried out using the pdb_mmcif70_14_Apr and NCBI Conserved Domains (CD) (v3.19) databases, accessed on 20 May 2022 (Table S4) (99, 100). The default parameters were used, i.e., the HHBlits UniRef30 MSA generation method, maximal generation steps = 3, and an E-value threshold of 1e−3. Minimum coverage of MSA hits was 20%, and minimum sequence identity of MSA hits with the query was 0%. Secondary structure scoring was done during alignment.

### Protein structure and function prediction.

Three-dimensional structures for the R. capsulatus RcGTA g16 protein was predicted using the AlphaFold colab server (https://colab.research.google.com/github/sokrypton/ColabFold/blob/main/beta/AlphaFold2_advanced.ipynb#scrollTo=bQe3KeyTcv0n) using msa_method:jackhammer and all other parameters set to default (45). The top-ranked AlphaFold model was submitted to the Dali protein structure comparison server to predict possible protein function; the full output can be found in Table S1.

### Data availability.

All data required to assess the findings are available in the main text or the supplemental information files. Reagents and materials used in this paper are publicly available. Any unique bacterial strains or recombinant DNA constructs described in this paper will be provided by the corresponding author upon request.
