# Supplementary material for: Loss of the Rhodobacter capsulatus Serine Acetyl Transferase Gene, cysE1, Impairs Gene Transfer by Gene Transfer Agents and Biofilm Phenotypes
Source: Appl Environ Microbiol. 2022 Sep 13;88(19):e00944-22. doi: 10.1128/aem.00944-22 (PMC9552610; doi:10.1128/aem.00944-22)
Supplement: Supplemental file 1 — Fig. S1 to S8. Download aem.00944-22-s0001.pdf, PDF file, 1.2 MB [file aem.00944-22-s0001.pdf]

Supplemental Figures

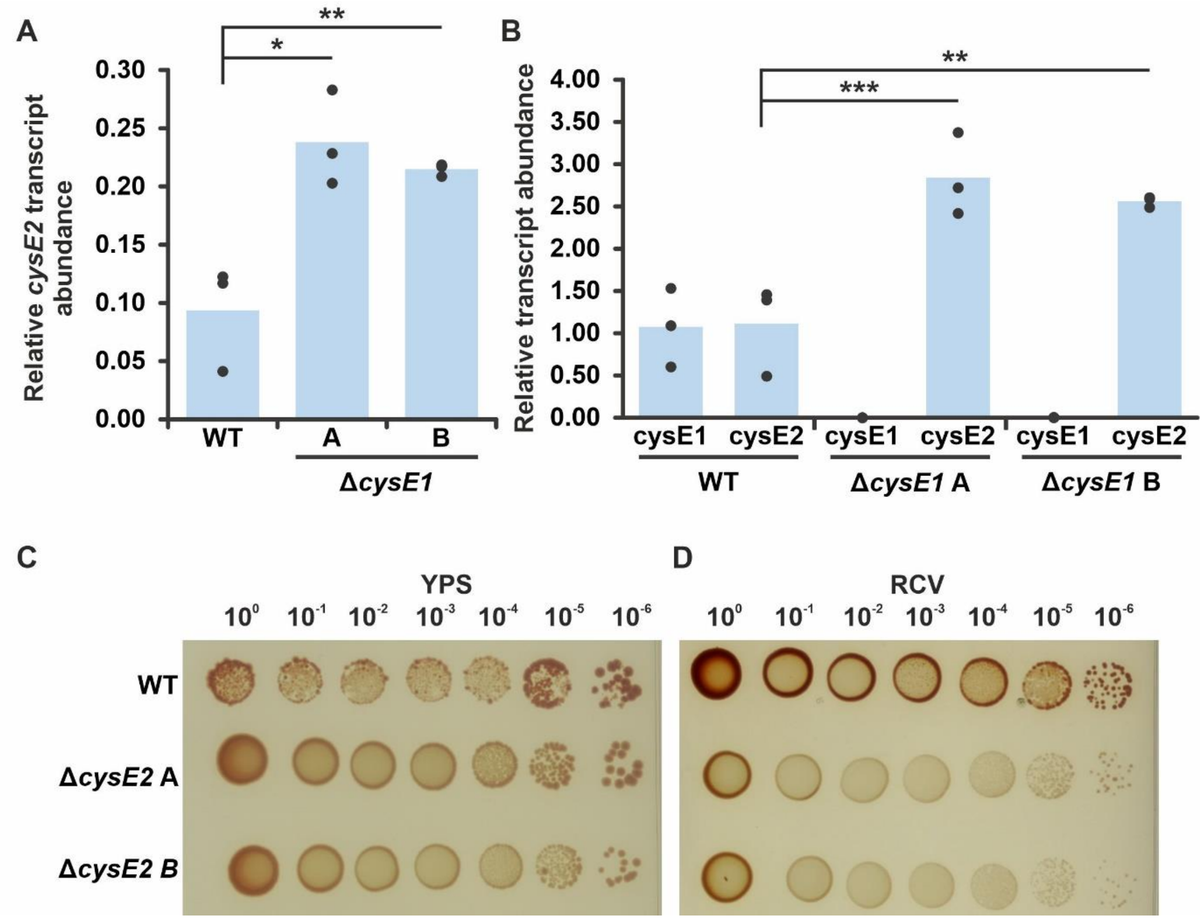

Figure S1. The *cysE2* gene is non-essential but may mitigate loss of *cysE1*. A. Relative expression of the *cysE2* gene in *R. capsulatus* WT and Δ*cysE1* mutants. Gene expression was measured by quantitative RT-PCR, and relative transcript abundance was determined by the ΔΔCt method. The transcription of *cysE2* was quantified in two independent *cysE1* deletions (A and B) and in the WT parental strain, relative to *cysE1* transcription in the parental strain. **B.** The same data from panel C was also used to assess *cysE1* and *cysE2* transcription in the wild-type *R. capsulatus* B10 parental strain and the two Δ*cysE1* strains, relative to the respective genes in the parental strain. In both cases, the *cysE1* deletion was confirmed and relative *cysE2* transcription was measured. Individual data points are plotted and bars represent the mean, n=3. Statistical significance is indicated above each chart (one-way ANOVA using the Holm-Sidak test; ***: $p<0.001$, **: $p<0.01$, *: $p<0.05$). **C & D.** Cultures of *R. capsulatus* SB1003 (WT) and two independent *cysE2* knock-outs (Δ*cysE2* A & B) were grown in RCV minimal media then subjected to a 10-fold serial dilution in RCV. Ten microliters of each dilution were dropped onto plates containing yeast extract and peptone (YPS) or minimal agar (RCV), as indicated.

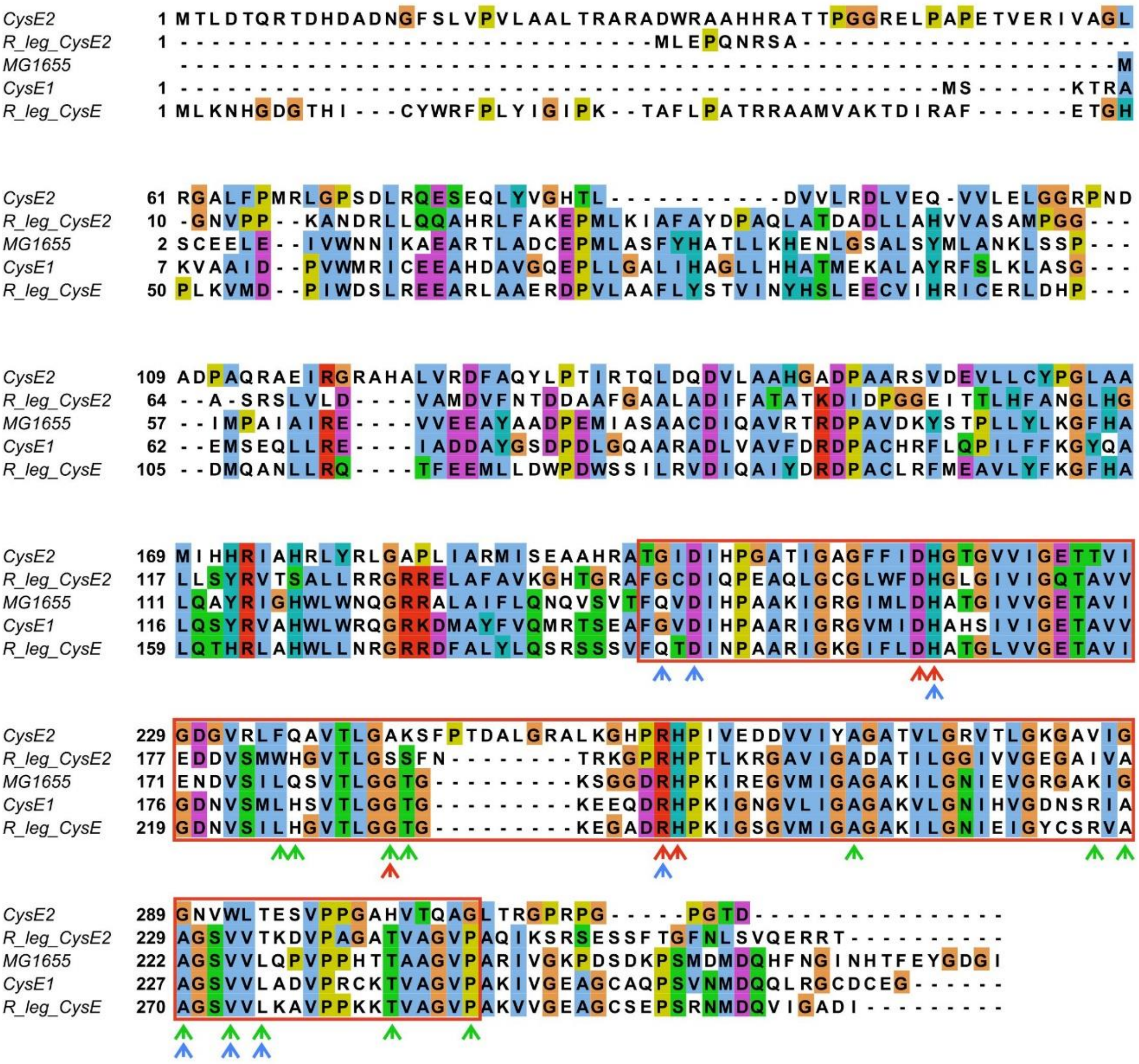

Figure S2. Comparison of *R. capsulatus* serine acetyl-transferase proteins with related sequences. The two *R. capsulatus* CysE proteins (CysE1 and CysE2) were aligned with counterparts from *Rhizobium leguminosarum* bv. *viciae* (R_leg_CysE, Accession: NKK74845.1; R_leg_CysE2, Accession: CAB70972.1) and *E. coli* K-12 CysE (MG1655, Accession: NP_418064.1). Alignments were created with Clustal Omega and visualized in JalView using the Clustalx homology colour scheme. Amino acid positions are labelled at the beginning of each row and gaps are indicated by dashes. The active site for the characterized *E. coli* CysE protein is indicated by a red box. Blue Arrows below the sequence indicate residues involved in trimer formation, Red Arrows indicate residues that bind the substrate i.e serine, green arrows indicate residues that bind Acetyl-CoA.

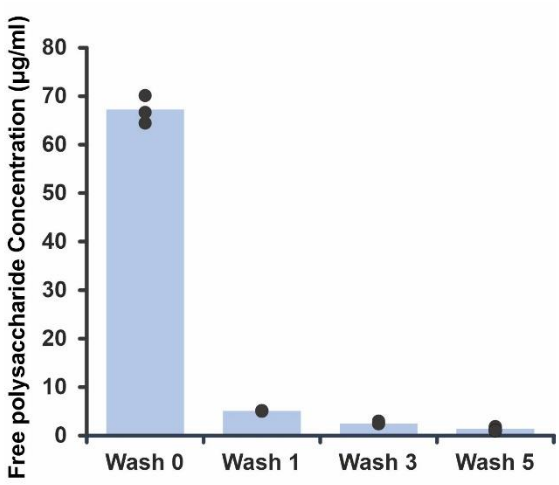

Figure S3. Free polysaccharides are present in the supernatant of wild-type *R. capsulatus* cultures. Polysaccharides were quantified directly in the cleared supernatant of wild-type *R. capsulatus* cultures (Wash 0) and after serial washes in 250 mM NaCl (Wash 1, 3, 5). Individual data points are plotted and bars represent the mean, n=3.

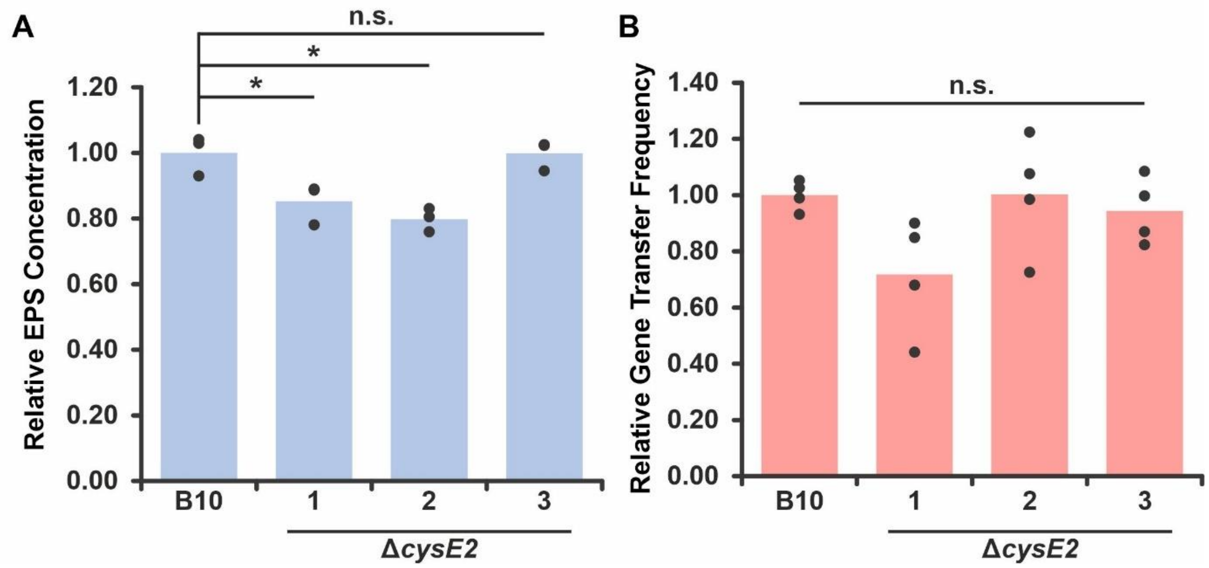

Figure S4. Deletion of *cysE2* has minimal effect on EPS production and GTA receipt by *R. capsulatus*. A. Relative concentration of cell-associated extracellular polysaccharide produced by three independent *cysE2* mutant lines (Δ*cysE2* #1-3) compared to wild type B10 cells. Individual data points are plotted and bars represent the mean, n=3. **B.** Relative frequency of GTA receipt by three independent *cysE2* mutant lines (Δ*cysE2* #1-3) compared to wild type B10 cells. Individual data points are plotted and bars represent the mean, n=4. Statistical significance is indicated above each chart (one-way ANOVA using the Holm-Sidak test; n.s.: no significance, *: $p<0.05$).

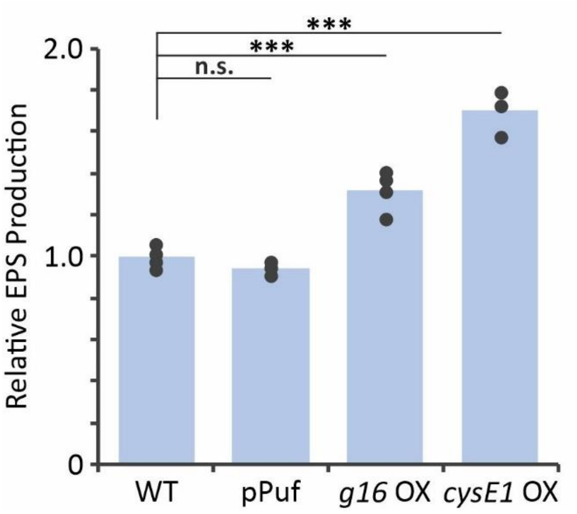

Figure S5. The effects of *g16* and *cysE1* overexpression on EPS production. Phenol:sulphuric acid quantification of the amount of cell-associated extracellular polysaccharide produced by the indicated overexpression strains relative to wild type. The pPuf control strain contains the *puf* promoter alone in pCM66T. Bars represent the mean and filled circles are the individual data points. Statistical significance is indicated above the chart (one-way ANOVA using the Holm-Sidak test, n=5; *: $p<0.05$, ***: $p<0.001$).

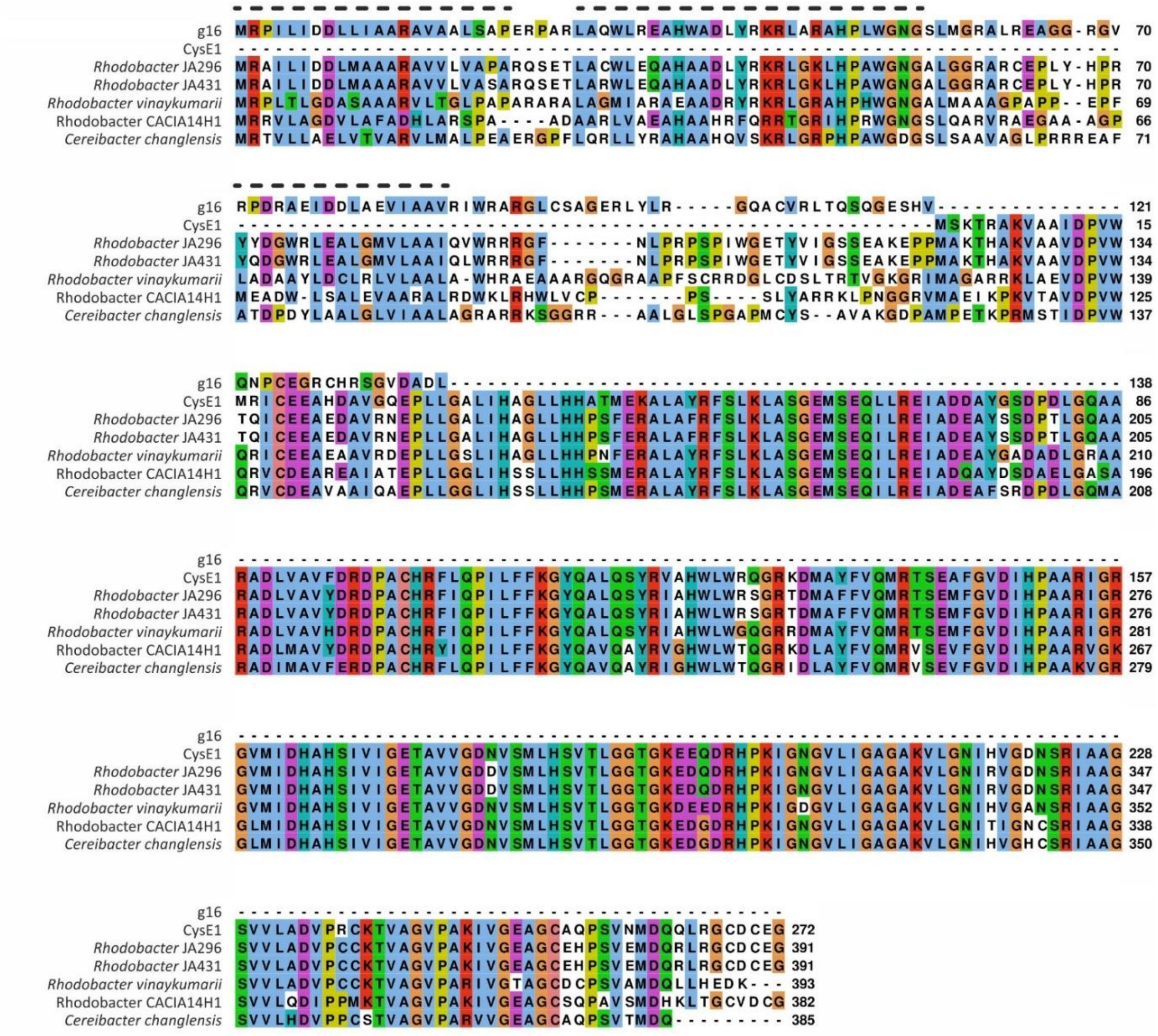

Figure S6. Alignment of *R. capsulatus* g16 and CysE1 proteins with five putative g16-CysE1 fusion proteins. Alignments were created with ClustalOmega and visualized in JalView using the Clustalx homology colour scheme. Amino acid positions are labelled at the end of each row and gaps are indicated by dashes. The g16 protein is less conserved than CysE1, but three regions of increased homology in the g16 region of the alignment are highlighted with dashed lines above the sequences. Protein accessions used in the alignment are *Rhodobacter aestuarii* JA296 (PTV97132.1) *Rhodobacter* sp. JA431 (SOB98634.1), *Rhodobacter vinaykumarii* (SIS49693.1), *Rhodobacter* sp. CACIA14H1 (ESW60554.1), *Cereibacter changlensis* (PZX57175.1).

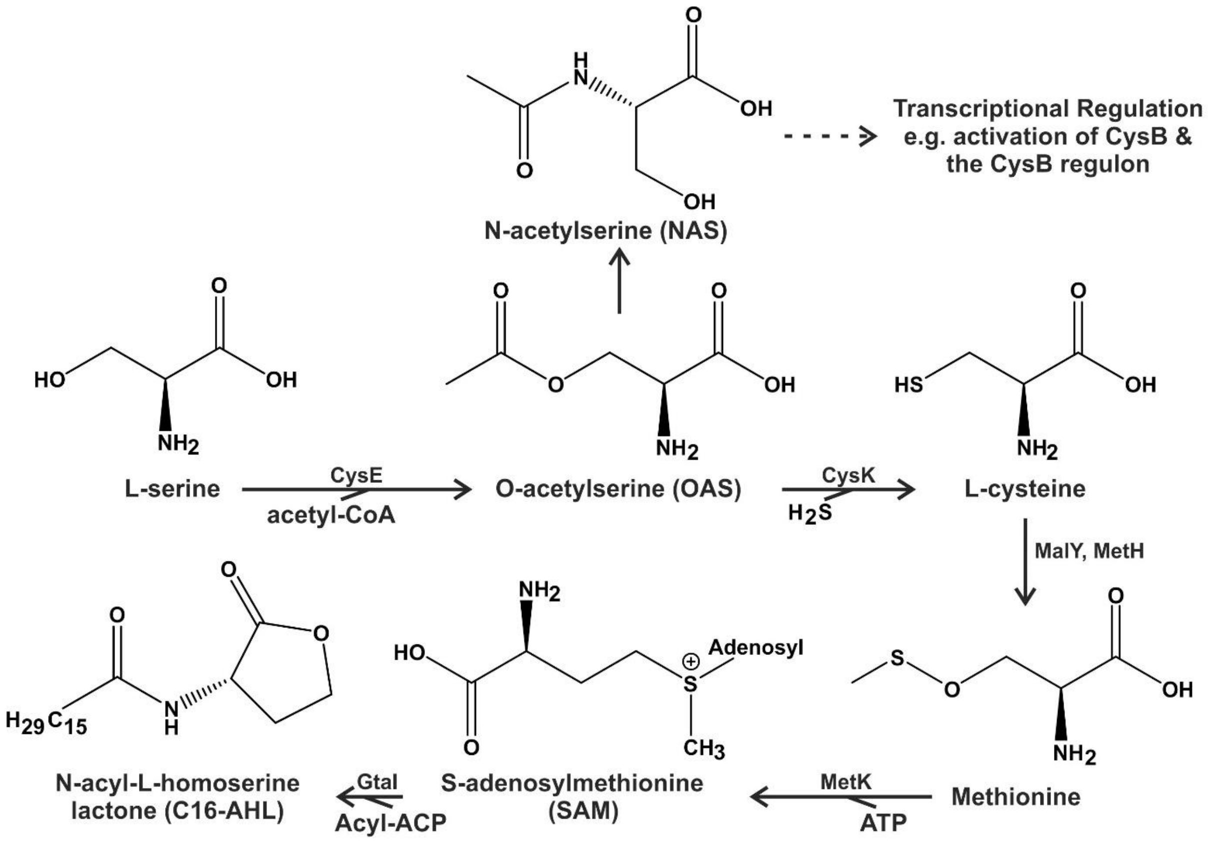

Figure S7. CysE-dependent biosynthesis pathway. CysE catalyses the first step of a biosynthesis pathway that has wide ranging effects including regulation of transcription via OAS and NAS, metabolism and replication via cysteine/methionine and cell-cell signalling via HSL. Catalytic steps are indicated by solid arrows with the enzymes for each step annotated immediately adjacent. Conversion of OAS to NAS occurs spontaneously. Dotted arrow indicates a regulatory signalling role rather than a catalytic reaction. ACP denotes acyl carrier protein.

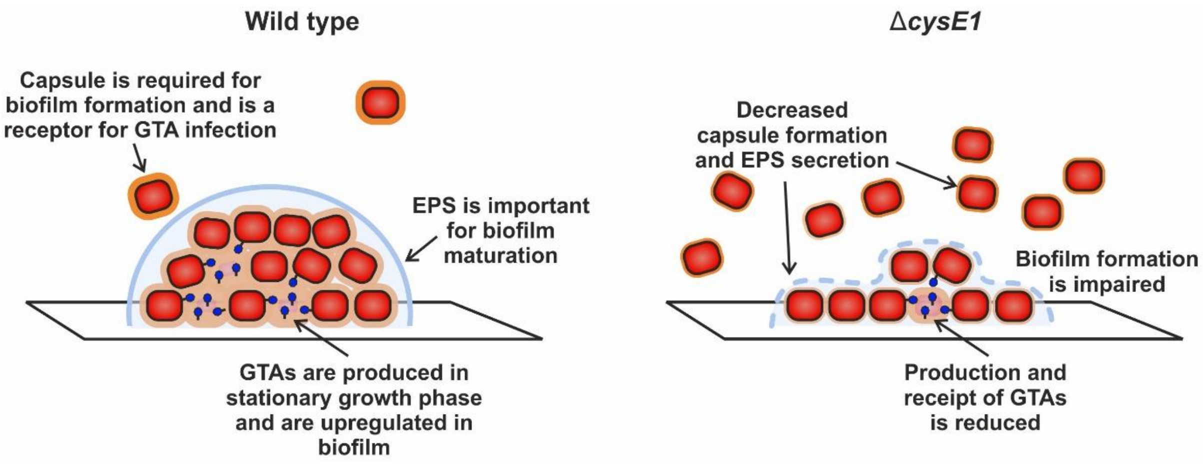

Figure S8. Schematic summary of proposed RcGTA production in biofilm.
